# Complete Genome Sequences of Virulent *Mycoplasma capricolum* subsp. *capripneumoniae* Strains F38 and ILRI181

**DOI:** 10.1128/genomeA.01041-14

**Published:** 2014-10-16

**Authors:** Laurent Falquet, Anne Liljander, Elise Schieck, Ilona Gluecks, Joachim Frey, Joerg Jores

**Affiliations:** a Biochemistry Unit, University of Fribourg and Swiss Institute of Bioinformatics, Fribourg, Switzerland; b International Livestock Research Institute, Nairobi, Kenya; c Vétérinaires Sans Frontières Suisse, Nairobi, Kenya; d Institute of Veterinary Bacteriology, University of Bern, Bern, Switzerland

## Abstract

Contagious caprine pleuropneumonia (CCPP) caused by *Mycoplasma capricolum* subsp. *capripneumoniae* is a severe epidemic affecting mainly domestic *Caprinae* species but also affects wild *Caprinae* species. *M. capricolum* subsp. *capripneumoniae* belongs to the “*Mycoplasma mycoides* cluster.” The disease features prominently in East Africa, in particular Kenya, Tanzania, and Ethiopia. CCPP also endangers wildlife and thus affects not only basic nutritional resources of large populations but also expensively built-up game resorts in affected countries. Here, we report the complete sequences of two *M. capricolum* subsp. *capripneumoniae* strains: the type strain F38 and strain ILRI181 isolated druing a recent outbreak in Kenya. Both genomes have a G+C content of 24% with sizes of 1,016,760 bp and 1,017,183 bp for strains F38 and ILRI181, respectively.

## GENOME ANNOUNCEMENT

In Kenya, goats account for more than 10,000,000 heads of livestock (1). Many resource-limited and livestock-dependent people, especially in semiarid areas, keep goats for their livelihood. Because of its high mortality, contagious caprine pleuropneumonia (CCPP) is one of the major diseases affecting goat, and to a lesser extent sheep, production systems. The causative agent is *Mycoplasma capricolum* subsp. *capripneumoniae*, a member of the so-called *M. mycoides* cluster that comprises five ruminant pathogens (2). CCPP also endangers wildlife, thus affecting not only basic nutritional resources of large populations but also income-generating game resorts and wildlife conservation in affected countries. Here, we report the genomes of two Kenyan strains—namely, the type strain F38 (NCTC 10192^T^) isolated in Kenya in the 1970s (3) and the highly virulent strain ILRI181 isolated from pleural fluid from a deceased adult goat from the Laikipia district in Kenya in 2012. This latter strain grew well on standard mycoplasma medium (Mycoplasma Experience, Ltd.). The genomic DNAs were isolated from bacterial cultures grown in liquid medium supplemented with serum. Sequencing was carried out at the NGS platform of the Universities of Bern and Fribourg on an Illumina HiSeq machine using standard protocols (100 bp PE). The resulting data were quality controlled with FastQC (http://www.bioinformatics.babraham.ac.uk/projects/fastqc) and assembled on the Vital-IT platform (http://www.vital-it.ch) using SPAdes software (4). Both strains had a raw coverage of about 200×. After running the first assembly, 16 contigs larger than 1,000 bp were obtained for ILRI181 and 5 for F38. The contigs were aligned to the genome of strain M1601 (5), and gaps were closed using targeted PCRs and manual correction of the draft assemblies. The final assembly resulted in circular genomes of 1,016,760 bp for F38 and 1,017,183 bp for ILRI181. We did not identify plasmids in both strains. The genomes were annotated using the Prokka pipeline (6) and deposited at the European Nucleotide Archive (7). Both genomes display 2 rRNA operons and 30 tRNA genes. F38 has 999 open-reading frames (ORFs), and ILRI181 has 998 ORFs, of which 435 are hypothetical in both genomes.

Genomic comparison of the sequences reveals a series of differences but less than 1,000 single nucleotide variants (SNVs) and a few indels. A genomic comparison of the available genomes, including the *M. capricolum* subsp. *capripneumoniae* strain Abomsa9231 isolated in Ethiopia in 1982 (8), confirms the very close similarity of available *M. capricolum* subsp. *capripneumoniae* genomes, as already indicated using multilocus sequence type data (8, 9). Interestingly, the most distant genome is the recent outbreak strain ILRI181. The genomes will enable genomic comparisons of the members of the *M. mycoides* cluster for the characterization of the pan and core genome and in order to enable reverse vaccinology approaches.

### Nucleotide sequence accession numbers.

Sequences were deposited at the European Nucleotide Archive under accession numbers LN515398 and LN515399 for the genomes of F38 and ILRI181, respectively.
